# The Importance of Being Taken Care of—Patients’ Experience with the Quality of Healthcare in a Norwegian Hospital

**DOI:** 10.3390/nursrep13040144

**Published:** 2023-12-15

**Authors:** Anne Karine Østbye Roos, Eli-Anne Skaug, Ann Karin Helgesen

**Affiliations:** 1 Intensive Care Department, Østfold Hospital Trust, P.O. Box 300, 1714 Grålum, Norway; 2 Faculty of Health, Welfare and Organisation, Østfold University College, P.O. Box 700, 1757 Halden, Norway; eli.a.skaug@hiof.no (E.-A.S.); ann.k.helgesen@hiof.no (A.K.H.)

**Keywords:** conventional content analysis, healthcare quality, patient perspective, hospital

## Abstract

As recipients of healthcare, patients provide invaluable contributions when assessing quality. The aim of this qualitative study was to explore and describe how patients experienced quality of care during their stay in a Norwegian hospital. In this study, 39 patients were interviewed. The data were analyzed via conventional content analysis. The results showed that patients’ experiences of quality were associated with interactions with medical staff, while their physical surroundings had less of an impact. The quality of healthcare was described on a continuum from good to bad. Patients assessed quality positively when they felt they were taken care of. The feeling of not being taken care of provoked feelings of insecurity, resignation, being “overlooked”, and inferiority. A prerequisite for patients to feel cared for was staff presence, which enabled and encouraged patients to share their thoughts. This required medical staff to have competency, interpersonal skills, and time accessibility. In addition, a culture that is person-oriented and not task-oriented was valued. From our study, we see the opportunity within healthcare systems to improve the quality of care by having staff engage in active listening, promote an environment of mutual respect, and encourage active participation from patients in their healthcare decisions and plans. This study was prospectively registered with the Norwegian Social Science Data Services on 9 July 2015 with registration number 44034.

## 1. Introduction

There is broad international consensus that measuring and evaluating patient experiences are important in the process of improving the quality of care in hospitals [1,2]. Having patients involved in treatment decisions is regarded as an international gold standard [3], and involving patients has also proved to have a positive impact on patient satisfaction [4]. A study by Rapport et al. showed that patients want to be informed, and a lack of information affects the opportunity to have their emotional needs met [5]. Fenton et al. argued that patients “bring expectations to medical encounters” and that addressing those needs is important [6]. While Fenton et al. [6] claimed that the level of patient satisfaction is connected to whether expectations are met, Grøndahl et al. [4] found that the level of patient satisfaction is related to whether a good relationship has been formed between the patient and healthcare personnel. This is in accordance with Park et al. [7], who described that patients’ experiences of quality were significantly linked to patients’ interactions with the staff. How patients experienced quality was, in particular, associated with good communication, as well as perceived support from nurses and the nurses’ attitude towards them. This finding is also in line with Woo and Choi [8], who found that the friendliness and consideration of the healthcare staff were the most important quality factors, as well as Ye et al. [9], who claimed that doctor and nurse communication was the most significant driver for patients’ experience of care.

How medical staff show compassion and respond to patients’ needs is a reflection of staff members’ interpersonal skills [5,10]. Those interpersonal skills can ultimately affect, both positively and negatively, a patient’s healthcare experience [5,10]. The relational aspects of care are reported by Kumah [1] to be an area that needs attention, and how staff use body language and courteous communication in patient encounters is of importance. Nevertheless, it is important to consider that the patients’ level of satisfaction is related to interactions with multiple healthcare providers [11], and Grøndahl et al. [12] described how the quality of care is related to both the environment and the surroundings, as well as the healthcare personnel.

In Norway, hospitals are required by law to assess and adapt their services based on feedback received from patients and their next of kin [13]. In the 2020 national user experience survey, the results showed that patients were most satisfied with the patient–nurse interaction, with the patient–doctor interaction, and with the information they received in conjunction with their hospital stay [14]. As recipients of healthcare, patients provide invaluable contributions to setting standards when assessing the quality of healthcare [15].

The aim of this study was to explore and describe how patients experienced quality of care during their stay in a Norwegian hospital. Although this study was conducted in a Norwegian setting, the results are transferable to other countries with similar healthcare organizations.

## 2. Materials and Method Design

This study had an exploratory and descriptive design. This design was chosen with the purpose of understanding the patients’ perceptions of the quality of care received during a hospital stay.

### 2.1. Setting

The study was conducted in a medium-sized public hospital in southern Norway. Most hospitals in Norway are public and are mainly government-funded. At the time of data collection, the hospital was about to relocate to a new building in another city with single bedrooms, bathrooms, higher standards, and advanced technological solutions. The old hospital was built with mostly shared bedrooms and bathrooms and to a low standard.

### 2.2. Entrance to the Field and Sample

This study is a sub-study of a larger project. Before the project was conducted, written permission was obtained from the heads of the hospital and the single departments. The nurse manager or the secretary in the unit delivered verbal and written information about the project to patients who were ready to be discharged from the hospital.

Certain criteria were set to include patients in the study. Participants were required to be 18 years of age or older, understand Norwegian, and be able to express themselves verbally. Prior to inviting patients to participate in the study, the nurses also assessed the patients’ mental and physical health to make sure participation was ethically justifiable.

The first inquiry for the patients focused on whether they would participate in a quantitative study that examined the patients’ perceptions of the quality of care. Patients who participated in the quantitative study were also asked if they could be contacted for in-depth interviews. From a total of 599 study subjects included in the quantitative study, 196 participants (33%) responded positively to being interviewed.

### 2.3. Informants

From the group of 196 participants that responded positively to being interviewed, a total of 40 patients were selected randomly and contacted by the second and third authors for in-depth interviews. Of the 40 informants who agreed to participate, 20 of the patients had stayed at the old hospital, and 20 of the patients had stayed at the new hospital.

One person who agreed to participate in the study subsequently withdrew due to illness. There were 19 women and 20 men included, ranging from 26 to 79 years of age, with a median age of 58 years. In total, 28 of the patients had been admitted to a medical ward, 6 to a surgical ward, 3 to a maternity ward, and 2 patients were unsure of which ward they had been admitted to.

### 2.4. Data Collection

The data were collected through individual interviews. A semi-structured interview guide was used. The interviews started and ended as open dialogues in which the participants were encouraged to talk freely about how they experienced the quality of care and what was most important to them during their hospital stay. Most interviews were conducted in the privacy of the patient’s home. The patients’ spouses were present in four of the interviews. Three interviews were conducted in a private office in a public building. The duration of the interviews ranged from 12 to 60 min, with a median length of 36 min. All interviews were recorded and transcribed verbatim. When the 39 included patients shared the same or similar information and comments, the second and third authors considered that data were saturated and information strength was good and that it was not necessary to perform further interviews.

### 2.5. Data Analyses

The data were analyzed through conventional content analysis in order to identify emerging themes and patterns. When there is limited existing theory or research on a specific phenomenon, conventional content analysis is an inductive method for a systematic examination of qualitative data [16]. The transcripts were read numerous times by all three authors in order to gain a better understanding of the situation as a whole. The main impression was written down, and the interview material was read verbatim by the second and third authors. Words and utterances considered meaningful (keywords) were marked. During the reading, when there were utterances that could be considered ambiguous, the audio recordings were played back to include the tone that would support the interpretations. Based on the marked words and utterances, codes for the meaning were established. The codes were compared and sorted, and categories and subcategories were established. The established codes and categories were discussed and adjusted by the author group until consensus was reached. Quotes were chosen to support the descriptions of the categories, thus strengthening the credibility of the results.

### 2.6. Ethical Considerations

The study was approved by the Norwegian Social Science Data Services (44034) and followed the principles of the Declaration of Helsinki [15]. The informants were informed verbally as well as given written information about the study. The information explained how data would be handled confidentially and that informants had the right to withdraw at any time without consequences. Informed consent was given by all informants.

The audio recordings, transcribed material, and consent forms were stored separately and locked away in the second author’s office. The audio recordings were deleted after the analysis of the data had been completed.

## 3. Results

One main category, “to feel cared for”, and the subcategories “to be acknowledged”, “to receive information”, and “staff presence” were derived from the data.

### 3.1. To Feel Cared for

The category shows that the feeling of being taken care of was crucial to patients’ descriptions of how they experienced the quality of the healthcare they received during their hospital stay. In order to feel as if they had been taken care of, interpersonal relationships were essential. Whether they had been admitted to the old or the new hospital had little significance for how patients described the quality of their experience. One patient with experience from staying in both the old and the new hospital expressed:

“The hospital was old and run down. The fact that it’s modern and simple is certainly nice, but I don’t think that is crucial to making you better any quicker. But I do value human relationships very highly.”

The quality of healthcare was described on a continuum from good to bad. Quality was often assessed positively when patients described how they felt taken care of. When things did not go according to plan, patients accepted and understood this, as long as they felt they were looked after. In cases where the patient did not feel as if they had been taken care of, the quality was assessed as negative. The feeling of not being taken care of provoked feelings of insecurity, resignation, being “overlooked”, and inferiority.

The categories “to be acknowledged”, “to receive information”, and “staff presence” represent different aspects of “to feel cared for”.

### 3.2. To Be Acknowledged

The category “to be acknowledged” consists of the aspects “to be seen and heard” and “to get help when needed”.

“To be seen and heard” was important for patients in order to feel they were being taken care of. Patients expressed that the staff did not always have to say much, but the feeling of being looked after was enough. To be acknowledged as an individual person was an important factor.

One patient was apprehensive about going in for surgery, and prior to this, she experienced not being seen or heard from the healthcare worker.

“I said aloud that I was dreading it, and she didn’t comment on it at all. She just continued talking, sitting in front of the computer, and said “Sit up there, I’ll have a look”, and so… She was so blunt and harsh; she wasn’t really… I felt like she didn’t see me at all.”

When staff did not recognize the patients’ feelings or help by addressing their concerns, patients expressed that they felt dehumanized and like a burden. Such experiences made it more difficult for patients to handle their situations, as one patient stated:

“If you’re met with… well, I don’t know, not much kindness or warmth, that’s what makes it harder to carry one’s weight.”

Patients described how illness and hospitalization intensified their need to be seen and heard. The feelings of being broken down and being small and frail made patients desire friendliness from the staff. It was also considered an important gesture that staff greeted the patient cordially upon entering the room, in contrast to just opening the door abruptly and claiming to be busy. Such acts added to the patients’ feelings of being a burden and did not add to the much-needed friendliness.

It was important for patients that the staff informed them of what they were going to do and recognized their needs. Otherwise, they felt overlooked and vulnerable.

One patient explained that the staff brought in the washbasin and the breakfast at the same time, which gave him the option of choosing between drinking a cold coffee or having a wash with cold water. The same patient also pondered: “They certainly have the knowledge. I wouldn’t say they didn’t know what they were doing, but the execution wasn’t professional at all.”.

To experience being acknowledged also included “to get help when needed”, for example, in relieving symptoms as well as having basic needs met. In our material, we found that patients had very different experiences about whether or not they got help when needed. Some patients described that they received the necessary help in prompt time, but some expressed the agony of having to wait for as long as an hour and a half to receive pain medication.

Not receiving the necessary help impacted the situation negatively, and the consequences of not getting help when needed were described as having a negative impact on the patient’s mental health.

### 3.3. To Receive Information

The category “to receive information” consists of how information is conveyed and how patients are informed about examinations and treatments.

Knowing what was going to happen added to the patient’s feeling of being safe and prepared. Patients receiving inadequate information experienced the situation as difficult and unsafe, as one patient stated, “*I was petrified. I wasn’t updated about anything.*”.

In addition to the uncertainty, a lack of information could create negative feelings and, as seen with this patient who was going in for surgery, an experience of inferiority:

“…, I had cried, I felt resigned, I was hungry, I was mentally worn out, my blood sugar was low, and my blood pressure wouldn’t go down…. I was even first on the list, but then I was put down to being number 2, and so I laid there waiting until midnight… But if I’d just known why. And I was also thinking, it happens… but why is what I’ll be going through not important? Is it not important? Am I not important? What’s going on that means I have to wait?”

How the patients perceived information could be seen in context with how the information was communicated. One patient described his experience of reading a booklet about his disease and seeing photos of the changes that happen to the internal organs as follows:

“Because I picked up all these booklets, but… so I’ll think I’ve got 11 new diseases from reading them, as I’m trying to understand… it’s this enlarged, and this is a heart muscle, and this is a fat deposit, and… I think… it should have been explained.”

It was significant that patients needed dependable and customized information, especially when technical languages or codes were used. Patients also mentioned that it was difficult to understand staff with a different mother tongue, proving that it is important that staff have sufficient language skills in order to communicate properly with patients.

### 3.4. Staff Presence

The category “staff presence” pertains to how patients perceive staff as accessible and how they have knowledge about the patients, their conditions, and their needs.

Staff accessibility was described on a continuum from “*They come as soon as you ring the alarm*” to “*They were not present at all*”. Experiences with staff members’ accessibility were described as good when agreements were kept and bad when they were not kept. Patients in our material also described that they felt overlooked when staff were preoccupied with computers.

The physical presence of staff was not always experienced as genuine presence, and this could generate feelings of not being valued, as one patient expressed:

“And my experience with the person who carried out the examination I was called in for was that he was a bit condescending and patronizing. He said a few things that I didn’t entirely understand, because he… didn’t speak all that clearly, and he spoke quickly as well, and wasn’t really… I felt that he wasn’t present.”

Patients described a positive experience when the staff members were familiar with their condition. The importance of staff informing each other about the patients’ situation was expressed by one patient:

“I felt that no matter who was on duty, they knew who I was and why I was there.”

Not everyone had such positive experiences, as one of the patients said:

“Whether or not they knew why I was there… I don’t think they did. They hadn’t read themselves up about me. No, I didn’t tell them about it (my story), and they didn’t seem like they were interested in it either.”

Finally, the patients valued the continuity of staff on a day-to-day basis during their hospital stay. Continuity of staff seemed to promote patient satisfaction and enhance patient–staff relationships. Patients who did not experience staff continuity expressed uncertainty and were dissatisfied. 

## 4. Discussion

The aim of this study was to explore and describe how patients experience quality of care during a hospital stay. Our results show that the patients’ experiences of quality were mainly linked to their interactions with medical staff and rarely to their physical surroundings.

Experience with quality was, therefore, not linked to whether the building was old or new. This result deviates from the quantitative research in the project, which showed a significant improvement in perceptions of quality when comparing the old hospital to the new hospital in terms of the physical environment [12]. Roos et al. [17] previously examined the experiences of patients staying in multiple-bed rooms compared to single-bed rooms. They found that single-bed rooms had advantages as they could better ensure the patient’s privacy as well as providing access to their own bathroom. At the same time, it was expressed that patients missed interpersonal contact in single-bed rooms when the lack of a common living room did not allow for contact with other patients. The need for contact with staff, thereby, increased.

In order to feel taken care of, it is essential that patients feel seen and heard. The importance of being acknowledged, seen, and heard as a person has been described in several studies [5,7,8,10,18]. It is the medical staff’s responsibility to establish such a relationship. Our results suggest that in order for this relationship to be experienced positively, the patient must feel that they are being heard, that their opinions are valued, and that they are respectfully acknowledged. In order for patients’ concerns to be taken seriously, medical staff must be familiar with the patient and understand the patient’s perception of their individual healthcare plan. Manary et al. [19] claimed that, when researching quality, an assessment must include the extent to which the patient and service provider had a common understanding. Such a common understanding can only be created when medical staff listen to the patient and acknowledge the received information while, at the same time, showing the patient respect and dignity. Whether patients experience being listened to or not depends on the staff’s behavior. How patients perceive being listened to by nurses has, so far, been little researched [20]. The informants in our study described that staff had both listening and non-listening behavior. One example of a staff member with non-listening behavior was a healthcare worker who was facing the computer instead of the patient during a preoperative conversation. Similar descriptions are also found in Loos’ study [20]. A common understanding is fundamental when it comes to providing personalized care and supplying adequate information when required.

The results of this study show that “receiving information” is important for patients. Communication, understood as sharing important information in an empathetic way and being treated with dignity, has previously been described as a core foundation for patients to have positive experiences [4,5,7,12,18,21,22]. Inadequate information will, therefore, have a negative impact on patient experiences and, thus, negatively influence the relationship between the medical staff and the patient. In previous studies, communication has proved to be the most essential factor when it comes to the patients’ experiences [10,23].

A study conducted by Rapport and Hibbert et al. [5] emphasized the importance of taking the patient’s concerns seriously. In our results, such consequences were described by one patient, who experienced a lack of personalized attention and care. This lack of personalized care led to the patient feeling small, frail, regarded as an object or a task, as a burden, and not as a person. The described feelings led to an aggravated situation for the patient, who claimed that this made it difficult for her to “carry her own weight”. It can be understood that this weakens the patient’s personal resources, or what Nightingale [24] describes as not facilitating the idea that nature’s innate forces can work or preserve the hope and courage necessary to handle the situation the patient finds themselves in. 

The informants’ descriptions of their negative experiences and the inadequate quality, such as the feelings of being overlooked and overwhelmed, lacking information, and not receiving help when needed, can be interpreted as examples of what Eriksson calls “suffering related to care” [25]. Not receiving help when needed, or a “lack of care from medical staff”, constitutes another type of suffering. A type of suffering related to care revolves around the exercise of power, which we observed in the story provided by a patient who was not being acknowledged by a healthcare worker. Furthermore, the healthcare’s subsequent examination was described by the patient as abrupt, harsh, and nonchalant. These unnecessary and inappropriate encounters can be considered a violation of the patient’s worth and dignity. The patient experienced the situation as if she were a task and not a person of value. Kumah [1] describes that respect and dignity are core values in clinical encounters between patients and healthcare professionals.

According to Eriksson [25], having staff that is present is a key component in eliminating this type of suffering. To see, to listen, and to offer help when needed require both presence and commitment. Medical staff must, therefore, be familiar with the patient and the patient’s perception of the care situation. Kumah [1] claims that this kind of interaction is a skill that staff can be equipped with through education. The responsibility for this relationship lies both with the medical staff and how the hospital is organized. Patients’ negative experiences can, therefore, be explained on both a systematic and individual level.

In an Australian study, patient experiences were shown to be particularly affected by the attitudes and behavior of the staff [10]. McCormack and McCance [26] state that seeing the individual and developing a relationship based on mutual trust are essential in person-centered practice. To understand and share knowledge based on respect for the individual and the individual’s rights are core elements [26]. According to the World Health Organization, patient-centeredness is a key dimension of healthcare quality [27]. It is also a prerequisite for collaboration and how patients participate in their own care and treatment [28]. A positive connection between the experience of patient-centered practice and quality is also documented in Edvardsson et al. [29] and Parlour et al. [30].

### Strengths and Limitations

The average age of the participants in this study was 58, and no one over the age of 77 participated. An overview from Statistics Norway showed that over 40% of admitted inpatients in hospitals were over 60, and 14% were over the age of 80 [31]. This study did not succeed in including the oldest age group, which can be considered a shortcoming.

There were two interviewers, and that may have influenced the data we obtained. We counteracted this by using a semi-structured interview guide that we prepared together and also by discussing in advance the purpose of the interview and current follow-up questions to the topics laid out in the interview guide.

## 5. Conclusions

In this study, we found that patients’ perceptions of the quality of healthcare received were primarily associated with whether they felt cared for and, thus, valued. In order to feel cared for, a key requisite is the presence of staff, which enables and encourages patients to share their thoughts. This requires medical staff to be competent, have interpersonal skills, and have adequate time accessibility. In addition to possessing competent and sufficient medical staff, a culture that is person-oriented and not task-oriented must be established. From our study, we see the opportunity within healthcare to improve the quality of care by having staff engage in active listening, promote an environment of mutual respect, and encourage active participation from patients in their healthcare decisions and plans. As the results in this study deviate from the quantitative research in the project, it proves how important it is to consider multidimensional approaches in order to understand patient experience. Further studies are needed to investigate possible additional communication training of medical staff and the opportunities for promoting a positive therapeutic cultural environment.

## Data Availability

The data are not publicly available due to confidentiality.

## Public Involvement Statement

No public involvement in any aspect of this research.

## Guidelines and Standards Statement

This manuscript was drafted against the CoreQ checklist https://www.equator-network.org, accessed on 7 September 2023.
